# Complete Genome Sequence of *Sulfuriferula* sp. Strain AH1, a Sulfur-Oxidizing Autotroph Isolated from Weathered Mine Tailings from the Duluth Complex in Minnesota

**DOI:** 10.1128/genomeA.00673-17

**Published:** 2017-08-10

**Authors:** Daniel S. Jones, Elizabeth W. Roepke, An An Hua, Beverly E. Flood, Jake V. Bailey

**Affiliations:** a University of Minnesota, BioTechnology Institute, St. Paul, Minnesota, USA; b Department of Earth Sciences, University of Minnesota, Minneapolis, Minnesota, USA

## Abstract

We report the closed and annotated genome sequence of *Sulfuriferula* sp. strain AH1. Strain AH1 has a 2,877,007-bp chromosome that includes a partial Sox system for inorganic sulfur oxidation and a complete nitrogen fixation pathway. It also has a single 39,138-bp plasmid with genes for arsenic and mercury resistance.

## GENOME ANNOUNCEMENT

Research on acidophilic sulfide mineral-oxidizing microorganisms from mining-impacted environments has transformed our understanding of acid rock drainage and led to advances in metal extraction and bioremediation of extremely acidic waters and mine waste (1–3). Less, however, is known about the microorganisms that are associated with circumneutral sulfidic waste rock and tailings. *Sulfuriferula* sp. strain AH1 was isolated from weathered mine tailings generated during pilot-plant processing of rock from copper-nickel deposits in the Duluth Complex, northeastern Minnesota (4, 5). The tailings were experimentally weathered for over 12 years using a humidity cell apparatus (6, 7), which generated leachate with pH values between 6.6 and 7.5 (4, 5). Culture-independent analysis revealed that *Sulfuriferula* spp. were some of the most abundant microorganisms in the humidity cell experiment (8).

Strain AH1 was enriched and isolated on solid thiosulfate medium [10 g Na_2_S_2_O_3_·5H_2_O, 1.5 g NaHPO_4_, 4 g KH_2_PO_4_, 0.4 g MgSO_4_·7H_2_O, 0.4 g (NH_4_)_2_SO_4_, and 0.04 g CaCl_2_·2H_2_O per liter, with a trace element solution (9) and 1.5% agar]. Strain AH1 is a member of the genus *Sulfuriferula* but is <97% similar by 16S rRNA gene sequence to the three described *Sulfuriferula* species (10–12).

Cells of strain AH1 were collected from batch cultures onto 0.2-μm-pore filters, and genomic DNA (gDNA) was isolated from the filters using the PowerWater DNA isolation kit (Mo Bio Laboratories, Inc.). Twenty micrograms of high-molecular-weight gDNA was used for long-read sequencing on a Pacific Biosciences RSII platform. SMRTbell libraries were prepared with the 20-kb insert size protocol at the Mayo Clinic Bioinformatics Core. PacBio reads were assembled in SMRT Analysis version 2.3 (13), according to the recommendations of Badalamenti et al. (14). The genome was initially assembled with HGAP version 3 (13) using a 10-kb minimum subread length cutoff. Circularity was confirmed by dotplot (15), contigs were manually circularized and reoriented, and the assembly was subsequently polished using all PacBio reads with Quiver (13) to >99.999% concordance (QV >50). This resulted in two circular contigs, a 2,877,007-bp chromosome at 303× coverage, and a 39,138-bp plasmid at 621× coverage. The genome was annotated with the JGI Integrated Microbial Genomes (IMG) Pipeline (16) and the NCBI Prokaryotic Genome Annotation Pipeline (17).

The genome contains 2,906 predicted protein-coding genes, 3 *rrn* operons, and 47 tRNAs (IMG annotation). Chemolithotrophic growth on reduced inorganic sulfur compounds is facilitated by features that include a partial Sox system (*soxXYZAB*) and a type I sulfide:quinone oxidoreductase (18). Strain AH1 has a complete Calvin-Benson cycle for carbon fixation, including two form I and one form II ribulose 1,5-bisphosphate carboxylase/oxygenase (RuBisCO) genes. One of the form I RuBisCOs occurs in an operon with genes for carboxysome synthesis. Other genomic features include a molybdenum-dependent nitrogenase and genes for flagellar assembly and chemotaxis. The plasmid contains *ars* and *mer* genes for arsenic and mercury resistance, respectively.

### Accession number(s).

The annotated chromosome and plasmid sequences are available in GenBank under accession numbers CP021138 and CP021139, respectively, and in the IMG database under genome ID 2716885007.
